# Mirror Mechanism Behind Visual–Auditory Interaction: Evidence From Event-Related Potentials in Children With Cochlear Implants

**DOI:** 10.3389/fnins.2021.692520

**Published:** 2021-08-24

**Authors:** Junbo Wang, Jiahao Liu, Kaiyin Lai, Qi Zhang, Yiqing Zheng, Suiping Wang, Maojin Liang

**Affiliations:** ^1^Department of Otolaryngology, Sun Yat-sen Memorial Hospital, Guangzhou, China; ^2^South China Normal University, Guangzhou, China; ^3^School of Foreign Languages, Shenzhen University, Shenzhen, China

**Keywords:** mirror mechanism, event-related potential, cochlear implant, hearing loss, speech performance, visual auditory

## Abstract

The mechanism underlying visual-induced auditory interaction is still under discussion. Here, we provide evidence that the mirror mechanism underlies visual–auditory interactions. In this study, visual stimuli were divided into two major groups—mirror stimuli that were able to activate mirror neurons and non-mirror stimuli that were not able to activate mirror neurons. The two groups were further divided into six subgroups as follows: visual speech-related mirror stimuli, visual speech-irrelevant mirror stimuli, and non-mirror stimuli with four different luminance levels. Participants were 25 children with cochlear implants (CIs) who underwent an event-related potential (ERP) and speech recognition task. The main results were as follows: (1) there were significant differences in P1, N1, and P2 ERPs between mirror stimuli and non-mirror stimuli; (2) these ERP differences between mirror and non-mirror stimuli were partly driven by Brodmann areas 41 and 42 in the superior temporal gyrus; (3) ERP component differences between visual speech-related mirror and non-mirror stimuli were partly driven by Brodmann area 39 (visual speech area), which was not observed when comparing the visual speech-irrelevant stimulus and non-mirror groups; and (4) ERPs evoked by visual speech-related mirror stimuli had more components correlated with speech recognition than ERPs evoked by non-mirror stimuli, while ERPs evoked by speech-irrelevant mirror stimuli were not significantly different to those induced by the non-mirror stimuli. These results indicate the following: (1) mirror and non-mirror stimuli differ in their associated neural activation; (2) the visual–auditory interaction possibly led to ERP differences, as Brodmann areas 41 and 42 constitute the primary auditory cortex; (3) mirror neurons could be responsible for the ERP differences, considering that Brodmann area 39 is associated with processing information about speech-related mirror stimuli; and (4) ERPs evoked by visual speech-related mirror stimuli could better reflect speech recognition ability. These results support the hypothesis that a mirror mechanism underlies visual–auditory interactions.

## Introduction

Visual–auditory interactions represent the interference between the visual system and the auditory system (Bulkin and Groh, 2006). A classic example of a visual–auditory interaction is the McGurk effect, in which video lip movements for [ga], dubbed by syllable sound [ba], lead to the auditory illusion of the fused syllable [da] (McGurk and MacDonald, 1976). This indicates that the visual system interferes with the auditory system. Another example is that pictures containing auditory information, such as “playing the violin” or “welding,” can activate the primary auditory cortex Brodmann area 41, while other pictures without auditory information do not (Proverbio et al., 2011). Furthermore, people with hearing loss show greater activation in the auditory cortex than normal-hearing controls during these kinds of stimuli; this suggests that the visual cortex compensates for hearing loss and could explain the basis of visual–auditory interactions (Finney et al., 2001; Liang et al., 2017).

Neuroimaging evidence has suggested that cross-modal plasticity might underlie visual–auditory interactions in patients with hearing loss (Finney et al., 2001; Mao and Pallas, 2013; Stropahl and Debener, 2017). This could indicate that a loss of auditory input causes the visual cortex to “take up” the auditory cortex *via* cross-modal plasticity and causes an interference of the visual system on the auditory system.

However, there are still some questions under discussion. For example, the McGurk effect is seen not only in patients with hearing loss but also in normal-hearing people, whose sensory tendency (a preference for visual or auditory interference) is no different to patients with hearing loss (Rosemann et al., 2020). Considering that cross-modal plasticity differs between these two groups while they showed no difference in visual–auditory interaction, there might be other mechanisms underlying visual–auditory interactions. We hypothesized that the mirror mechanism is one such mechanism underlying this visual–auditory interaction.

First, neurons that discharge in response to both observation and execution are called mirror neurons, and the theory to explain their functions is called mirror mechanism. For example, it has been reported that a large proportion of a monkey’s premotor cortical neurons discharge not only while performing specific actions but also when hearing associated sounds or observing the same actions (Keysers et al., 2002); thus, in observational processes, mirror neurons imitate what they do in executional processes. Incidentally, visual–auditory interactions are associated with a similar phenomenon, whereby the auditory cortex imitates what it does in response to auditory stimuli during the presentation of visual stimuli.

Second, mirror mechanism allows “hearing” to be an executional process. One ongoing issue is that hearing is a feeling rather an executable action and thus cannot be classified as a “motor action,” which refers to the executional process of the mirror mechanism. However, some researchers have argued that “motor actions” encoded by the mirror mechanism are actually “action goals” rather than muscle contractions or joint displacement (Rizzolatti et al., 2001; Rizzolatti and Sinigaglia, 2016). For example, mirror mechanism has been related with empathy (Gazzola et al., 2006) and animal sounds that were mistakenly recognized as tool sounds, thus activating similar cortical areas as the tool sounds (Brefczynski et al., 2005); empathy and sound recognition are not muscle contractions or joint displacement. Currently, mirror mechanism has been defined as “a basic brain mechanism that transforms sensory representations of others’ behavior into one’s own motor or visceromotor representations concerning that behavior and, depending on the location, can fulfill a range of cognitive functions, including action and emotion understanding”; furthermore, “motor actions” usually refer to the outcomes induced by actions rather than the actions themselves (Rizzolatti and Sinigaglia, 2016). Thus, mirror mechanism might allow “hearing” to be an executional process.

Third, we have consistency of activated nerve localization in visual–auditory interaction and mirror neurons. One experiment reported that visual–auditory stimuli can activate mirror neurons (Proverbio et al., 2011), but has not been further discussed. This might be due to limitations that are inherent to the study design, whereby pictures with no auditory information are complex and may therefore cause activation of mirror neurons.

To conclude, it is reasonable to suspect that the mirror mechanism might underlie visual–auditory interactions. In this study, we measured event-related potentials (ERPs) and speech recognition ability in children with cochlear implants (CIs) during mirror neuron-activated and mirror neuron-silent visual–auditory interaction tasks. We aimed to determine the role of this mirror mechanism in visual–auditory interactions.

## Materials and Methods

### Participants

Twenty-five prelingually deaf children fitted with a CI for at least 4 years were recruited. These included 12 boys and 13 girls aged between 5 and 18 years (mean: 9.86; SD: 3.80). Table 1 presents the detailed demographic information. All participants were right-handed and had normal or corrected-to-normal vision. Ethical approval was obtained from the Institutional Review Board at Sun Yat-sen Memorial Hospital at Sun Yat-sen University. Detailed information was provided to the parents, and written consent was obtained before proceeding with the study.

**TABLE 1:** Information of all participants in this study.

Number	Gender	Age at experiment(y)	CI side	Age at CI(y)	Age at hearing loss(y)	Speech test
1	Male	7	Right	1	0	No
2	Male	8	Left	3	2	No
3	Male	13	Right	5	0	No
4	Female	16	Right	3	0	No
5	Male	11	Right	2	0	Yes
6	Female	9	Right	5	4	Yes
7	Male	13	Right	10	1	No
8	Female	7	Right	3	0	Yes
9	Female	8	Right	4	2	Yes
10	Male	7	Right	2	0	Yes
11	Male	7	Right	2	0	Yes
12	Female	10	Both	4	1	No
13	Male	7	Right	1	0	Yes
14	Female	18	Right	6	1.5	No
15	Male	7	Right	2	6	No
16	Female	6	Right	1	0	No
17	Female	9	Right	3	1.4	No
18	Female	9	Right	3	2	No
19	Male	10	Right	2	0	No
20	Female	17	Right	3	0	Yes
21	Female	6	Right	2	0	No
22	Female	18	Right	13	4	Yes
23	Male	9	Left	2	1	Yes
24	Male	9	Right	2	Unknown	No
25	Female	7	Right	1	0	Yes

### Speech-Related Behavioral Tests

Of the 25 participants, 11 took part in the behavioral experiment. The behavioral experiment comprised six tasks as follows: easy tone, difficult tone, easy vowel, difficult vowel, easy consonant, and difficult consonant. For the experimental materials and classification standards, we referred to *Research on the Spectrogram Similarity Standardization of Phonetic Stimulus for Children* (Xibin, 2006). Vowels were classified according to the opening characteristics and internal structural characteristics of the first sound. As a result, vowel recognition can be divided into four groups according to these two dimensions, namely, the same structure with different openings, the same opening with different structures, the same opening with the same structure, and the compound vowels of the front nose and the rear nose. Consonants can be classified into six groups according to the two dimensions of pronunciation position and pronunciation mode, namely, fricative/non-fricative recognition, voiced consonant/clear consonant recognition, aspirated/non-aspirated consonant recognition, same position/different consonant recognition, rolled tongue/non-rolled tongue sound recognition, and same position/different consonant recognition (Note: The recognition of/z/zh/,/c/ch/, and/s/sh/fall under the recognition groups of flat tongue sound and crooked tongue sound. Given that the stimulation is very close to the pronunciation part, it is also very difficult for the normal-hearing population, so it is classified as a separate group as the most difficult content.).

Using the syllable as the task unit, recognition difficulty was defined to control for its different effects between consonants and vowels, in the tone discrimination task, and consonants within syllables of the same category, and pure in the plosives. Vowels only included the monophonies of/a/,/e/,/i/,/o/, and/u/. In the easy tone task, subjects were asked to distinguish between the first tone and the fourth tone, which are evidently different; in the difficult tone task, subjects were asked to distinguish between the third tone and the fourth tone, which are more similar to each other. There were 80 syllable pairs presented in the easy and difficult tone discrimination conditions, and the task contained 32 “same” and 48 “different” trials.

In the consonant recognition task, as mentioned above, we divided consonants into six groups according to the two dimensions. To reduce the effect of the syllable and vowel, the syllables in each group contained four tones, and the vowels in each group were monosyllabic. The difficulty of a consonant discrimination task was determined by the similarity of the two consonants. Those with a large difference in consonant type were classified as easy, while those with a small difference were classified as difficult. For example,/h/and/m/are different in pronunciation position and pronunciation mode, while/h/and/k/are only different in pronunciation mode; thus,/h/and/m/are classified as belonging to the easy recognition group, while/h/and/k/are classified as belonging to the difficult recognition group. There were a total of 100 syllable pairs in the consonant recognition task, with 40 “same” and 60 “different” trials.

In the vowel recognition task, vowels were divided into four groups according to the two dimensions. The syllables of each group contained four tones. Easy vowel recognition trials contained vowels from different vowel groups, and difficult vowel recognition trials included compound vowels with more similarity, thus making it harder to distinguish syllables. The vowel discrimination task included 60 easy trials, namely, 36 different stimulus pairs and 24 same stimulus pairs. There were 100 trials, which included 40 “same” stimulus pairs and 60 “different” stimulus pairs.

### ERP Measurement

#### Non-mirror Stimuli

All children underwent ERP measurements. We adopted visual stimuli consisting of reversing displays of circular checkerboard patterns created by Sandmann et al. (2012), which have been used to examine cross-modal reorganization in the auditory cortex of CI users. There were four different pairs of checkerboard patterns, which systematically varied by the luminance ratio. The proportions of white pixels in the stimulus patterns were 12.5% (Level 1), 25% (Level 2), 37.5% (Level 3), and 50% (Level 4) (Supplementary Figure 1). The contrast between white and black pixels was identical in all images used.

Subjects were seated comfortably in front of a high-resolution 19-inch VGA computer monitor at a viewing distance of approximately 1 m, in a soundproof and electromagnetically shielded room. All stimuli were presented *via* E-prime 2.0, and stimulus software was compatible with Net Station 4 (Electrical Geodesics, Inc.). The checkerboard stimulus remained on the monitor for 500 ms and was immediately followed by blank-screen with an inter-stimulus interval of 500 ms. Each presented blank stimulus image included a fixation point (a white cross) at the center of the screen. Participants performed four experimental blocks (i.e., conditions), in which they were presented with one of the four image pairs. The block order was counterbalanced across participants. During the experimental session, each checkerboard image was repeated 60 times, resulting in a total of 480 stimuli (4 conditions × 2 images × 60 repetitions). Participants were instructed to keep their eyes on the pictures before each condition and could rest for over 1 min between blocks.

#### Mirror Stimuli

In this study, the mirror stimulus is a visual stimulus with certain behavioral information, thus they are able to active mirror neurons. Based on the relationship of behavioral information to speech, mirror stimulus could be further divided into speech-related mirror stimulus (i.e., a photograph of the action of reading) and speech-irrelevant stimulus (i.e., a photograph of the action of singing). Considering that current CIs cannot faithfully relay musical rhythm (Kong et al., 2004; Galvin et al., 2007; Limb and Roy, 2014), the picture of singing might just mean the movement of mouth to CI children. However, we will still use the term “singing” to describe the action in this paper. Visual stimuli were presented in a similar way to those in the study by Proverbio (Proverbio et al., 2011). These stimuli have been found to induce visual–auditory interactions in a previous study (Liang et al., 2017). Photographs that most of the children were familiar with and the content of which was understood were chosen. Supplementary Figure 2 shows the experimental block design, which consisted of an intermittent stimulus mode using speech-related mirror stimuli and speech-irrelevant mirror stimuli. To measure ERPs elicited by speech-related mirror stimuli, the experiment consisted of 85 trials of the “reading” photo stimuli and 15 trials of the “singing” photo stimuli as deviant stimuli. In contrast, to measure ERPs elicited by speech-irrelevant mirror stimuli, the experiment consisted of 85 trials of the “singing” photo stimuli and 15 trials of the “reading” photo stimuli as deviant stimuli. As shown in Supplementary Figure 2, each stimulus was presented for 1 s, followed by a blank screen (1.7–1.9 s in duration) as the inter-stimulus. To ensure that participants remained focused on the stimuli, one novel trial of 15 photographs was presented after 5–10 trials, in response to which children were asked to press a button when they saw the deviant photograph.

### EEG Recording

Electroencephalography (EEG) data were continuously recorded using a 128-channel EEG electrode recording system (Electrical Geodesics, Inc.). The sampling rate for the EEG recording was 1 kHz, and electrode impedances were kept below 50 kΩ. For ERP analyses, the data of individual participants were band-pass filtered offline at 0.3–30 Hz and segmented into epochs of 100 ms pre-stimulus and 600 ms post-stimulus. Artifact rejection set at 200 mV was applied to visual EEG, and epochs were rejected if they contained any eye blinking (eye channel exceeded 140 mV) or eye movement (eye channel exceeded 55 mV). Bad channels were removed from the recording. Data were then re-referenced using a common average reference. The data were baseline-corrected to the pre-stimulus time of −100 to 0 ms.

Amplitudes and latencies of the P1–N1–P2 complex on the 75(Oz) electrode for individual participants were analyzed. The time window was set to 90–180 ms for the P1, 110–320 ms for the N1, and 220–400 ms for the P2. The amplitudes of the P1, N1, and P2 were measured as the baseline to peak value. Individual subject latencies were defined using the highest peak amplitude for each visually evoked potential. Individual waveform averages were averaged together for each group to compute a grand-average waveform.

### Statistical Analysis

For statistical analysis, groups were divided by the various types of stimuli as follows: speech-related mirror stimuli, speech-irrelevant mirror stimuli, non-mirror stimuli level 1, non-mirror stimuli level 2, non-mirror stimuli level 3, and non-mirror stimuli level 4. An ANOVA was applied to examine between-group differences in ERP components. The Least Significant Difference test was then used to determine from which group–group comparison the significant difference originated.

Applying standardized low-resolution brain electromagnetic tomography (sLORETA) (Fuchs et al., 2002; Pascual-Marqui, 2002; Jurcak et al., 2007), a source location analysis was performed for the ERP components (P1, N1, and P2). The point in ERP (such as group A vs. group B) we chose to measure the difference in latency was calculated from the mean of group A and group B {[Latency (A) + Latency (B)]/2}. The top five brain regions in which there was a difference were revealed.

The assumption of a normal distribution was not satisfied, and so Spearman’s correlation coefficient was used to test the correlations between ERP components (amplitude and latency included) and speech behavior. For all tests, a *p*-value < 0.05 was taken to indicate significance.

## Results

### Event-Related Potentials

#### Between-Group Differences

**FIGURE 1:**
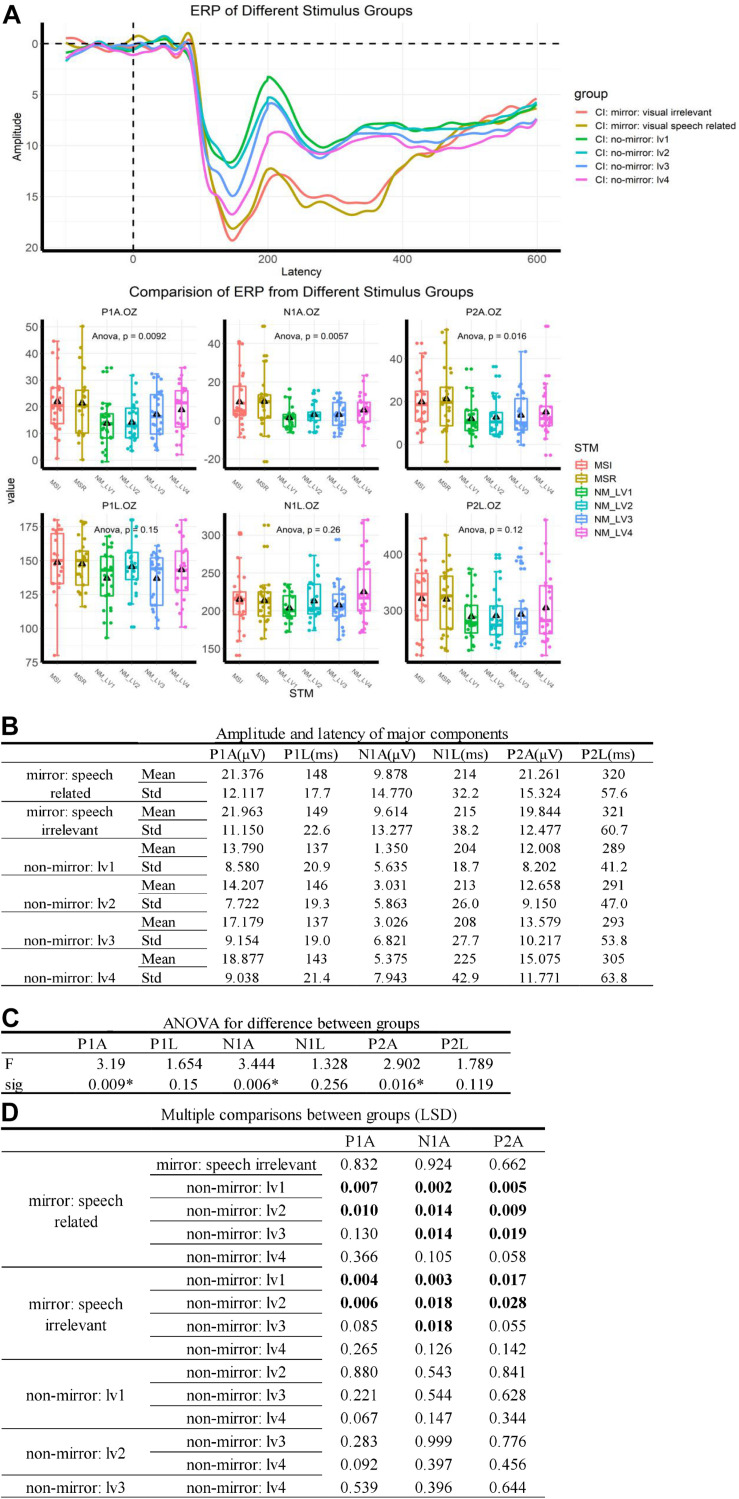
ERP results. **(A)** ERP traces for the different groups. Stimuli were delivered at 0 ms, and a P1–N1–P2 complex was observed after 100 ms. MVSR: mirror and visual speech-related, MVSI: mirror and visual speech-irrelevant, NM: no-mirror. Delta: mean value. **(B)** Values used to describe ERP components. **(C)** An ANOVA showed that the difference between groups was mainly caused by differences in ERP amplitudes. ^∗^*p* < 0.05. **(D)** Multiple comparisons post-ANOVA. “Boldface number” means *p* < 0.05, indicating that significant differences in the ANOVA were mainly caused by the difference between the mirror stimulus group (including speech-related mirror stimuli and speech-irrelevant stimuli) and the non-mirror stimulus group (including the four types of non-mirror stimuli).

Event-Related Potentials results are summarized in Figure 1. Significant differences were observed in the P1, N1, and P2 components between ERPs evoked by mirror stimuli and those evoked by non-mirror stimuli (Figure 1C). The data from normal-hearing controls are shown in Supplementary Figure 1. In a word, in the two major stimulus groups, ERPs elicited by the mirror stimuli differed significantly to the non-mirror stimuli. However, in subgroups, no significant difference in ERPs was found between the speech-related mirror stimuli and speech-irrelevant mirror stimuli, nor between the four types of non-mirror stimuli. Data for the normal-hearing control group are shown in Supplementary Data.

#### Source Location of ERP Differences

**FIGURE 2:**
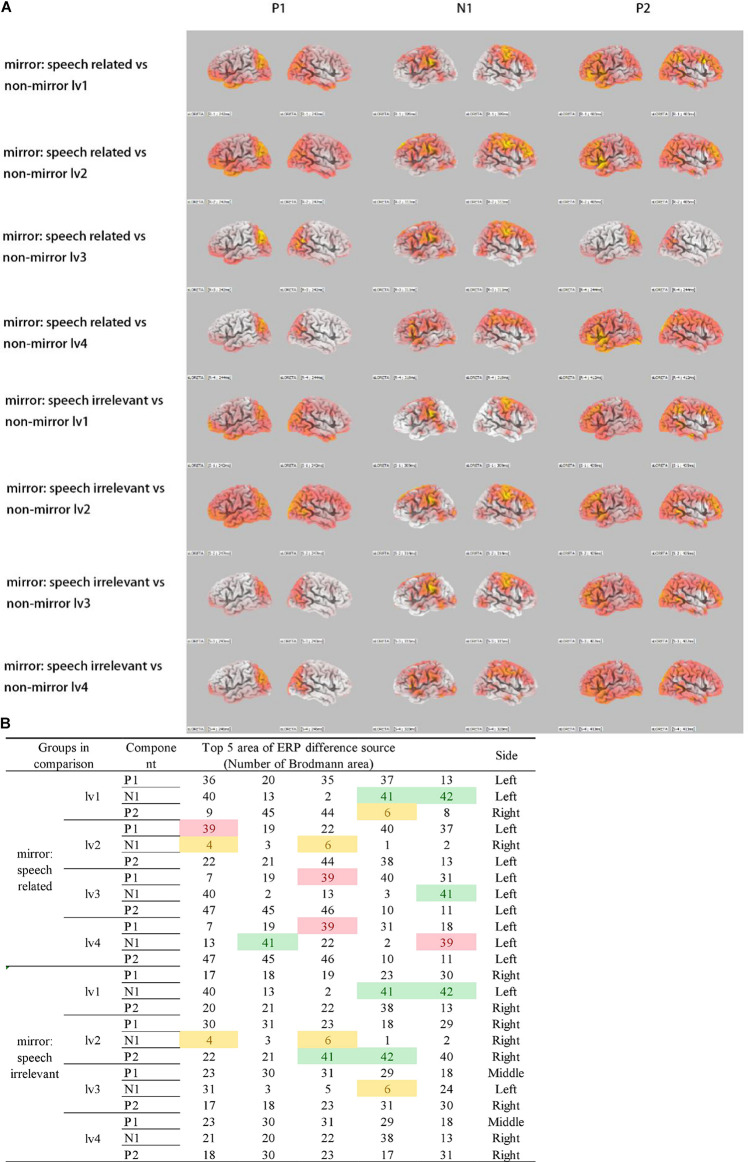
Source location of ERP differences. **(A)** sLORETA for ERP differences between groups. Colorful regions indicate the source of ERP differences. **(B)** Red: BA39 (left), which is the visual speech cortex. Green: BA41 and BA42, which make up the primary auditory cortex. Yellow: BA4 and BA6, which make up the precentral gyrus. Only speech-related stimuli activated BA39 differently, compared with non-mirror stimuli.

An sLORETA analysis was applied to the ERP data to assess between-group differences, and the results are shown in Figure 2.

On comparing ERPs between the non-mirror and mirror stimuli groups, the primary auditory cortex in the superior temporal gyrus, including Brodmann area 41 (B41) and BA42, was strongly activated for the mirror stimuli, which indicates the presence of a visual–auditory interaction. Furthermore, the precentral gyrus, including BA4 and BA6, was more strongly activated in the mirror stimuli vs. the non-mirror stimuli groups. Moreover, compared with ERPs elicited by the non-mirror stimuli, BA39, the visual speech area in the angular gyrus was strongly activated only for the speech-related mirror group. This was not observed when comparing ERPs between the non-mirror stimuli and speech-irrelevant mirror groups.

#### Correlation Between ERP Component and Speech Recognition

**FIGURE 3:**
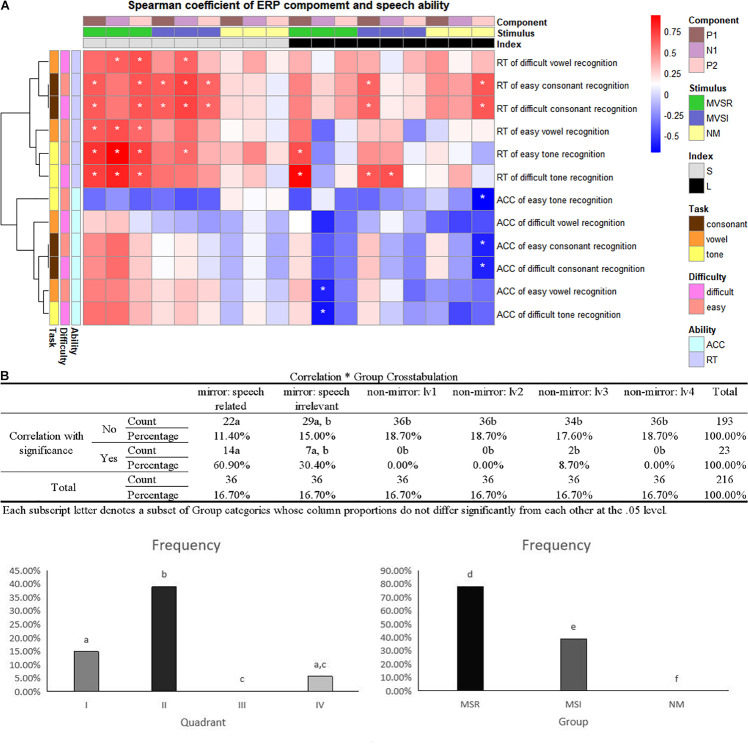
Correlation between ERP component and speech recognition. **(A)** Heatmaps of Spearman’s correlation coefficient (–1, +1). ACC, accuracy; RT, reaction time; MVSR, mirror and visual speech-related; MVSI, mirror and visual speech-irrelevant; S, stimulus peak; L, latency; ^∗^*p* < 0.05. **(B)** As the heatmap is a combination of ERP components and speech measures, we tried to find which type of combination (for example: ERP latency–task RT performance vs. ERP peak–task RT performance) resulted in stronger correlations by calculating the percentage of cells with “^∗^” in all cells of specific combination. Left: four quadrants as four combinations; right: in II quadrant, MSR/MSI/NM peak–RT performance as three combinations. (I, II, III, IV: quadrant of the heatmap, I: latency–task RT performance, II: peak–task RT performance, III: peak–task ACC performance, IV: latency–task ACC performance. a, b, c, d, e, f: in analysis of crosstab, the same character indicates no significant difference, while a different character indicates a significant difference).

Correlation analysis showed that the combination of stimulus ERP peak–task RT performance enriches most significant correlations (Figures 3A,B, left). Further crosstab analysis revealed that the combination of ERP peak in mirror and visual speech-related (MVSR)–task reaction time performance includes most significant correlations within the combination of ERP peak–task RT performance. This indicates that, compared with the “mirror: visual speech-irrelevant” and “no-mirror” stimuli, the “mirror: visual speech-irrelevant” stimuli induced ERPs that better reflected speech task performance.

## Discussion

### Explanation for the Results

We hypothesized that the mirror mechanism underlies visual–auditory interactions. Concerning the visual–auditory interaction element, mirror stimuli denoting reading and singing actions were able to induce visual–auditory interactions, while the non-mirror stimuli did not. People with hearing loss tend to receive speech-related information indirectly, such as *via* lip reading (Geers et al., 2003; Burden and Campbell, 2011). Also, rehabilitation training in those with hearing loss encourages reading loudly; while the sounds may not be accurate, this may activate the auditory cortex. Singing is obviously sound-related, while a black–white checkerboard is not related to sound. This could explain why the two major stimulus groups (mirror vs. non-mirror groups) induced different ERPs, which was driven by the primary auditory cortex (BA41and BA42).

Concerning the element of the mirror mechanism, mirror stimuli showing reading and singing might activate mirror neurons, unlike non-mirror stimuli. Since mirror stimuli that evoke this mirror mechanism should have the same action goal while both observing and executing the process, stimuli with an imitable action are likely to activate mirror neurons; this interpretation is consistent with previous studies (Kohler et al., 2002; Vogt et al., 2004; Michael et al., 2014). In contrast, a checkerboard image is much less likely to activate mirror neurons, as the function of mirror mechanism is to benefit the individual by imitating actions, comprehending actions, and understanding feelings; a meaningless geometric figure fits none of these functions. This could explain why the two major stimulus groups evoked different neural activities, reflected in ERP differences.

Spatial source of ERP difference is located in mirror neurons. According to the mirror mechanism theory, a mirror neuron can be activated during both execution and observation of an action. In this context, both the action and perception of reading will activate the visual speech area (BA39). To be classified as a mirror neuron, the neuron needs to be activated in both the observation and execution of reading. For singing, our region of interest was the motor cortex, including BA4 and BA6.

Previous works have noted that visual–auditory interactions differ between normal-hearing people and those with hearing loss (Stropahl and Debener, 2017; Rosemann et al., 2020), and that visual–auditory interactions might be related to speech performance in children with a CI (Liang et al., 2017). This indicates that visual–auditory interactions are related to speech comprehension in people with hearing loss. Considering the ability of mirror neurons to encode motor actions (di Pellegrino et al., 1992; Gallese et al., 1996; Rizzolatti et al., 1996), the relationship between visual–auditory interactions and speech recognition ability might be related to this ability. This could explain why the speech-related stimuli evoked stronger visual–auditory interactions that are reflected by speech recognition ability.

### ERP Results

First, ERP results revealed that visual–auditory interactions were induced in the mirror stimuli groups, whether the stimuli were speech-related or irrelevant, with the strongest activation in the primary auditory cortex (Figure 2). Our finding of the primary auditory cortex activation is consistent with those of a previous study, in which the primary auditory cortex (BA41) was activated by sound-related photos at around 200 ms after stimulus presentation (Proverbio et al., 2011).

Second, on comparing the ERPs elicited by the mirror stimuli and non-mirror stimuli, the results indicated that the mirror neurons were more strongly activated in the “motor action” condition than in the functional cortex. More precisely, an image of reading evoked a stronger activation in the visual speech area (left BA39; Figure 2), while the image of singing induced a stronger activation in the precentral gyrus (BA4 and BA6), where the mouth and hand areas are located (Figure 2).

Third, we found that that difference in the degree of checkerboard lightness did not significantly affect ERPs. However, a trend was found in the effect of luminance on ERPs, which is consistent with a previous study (Sandmann et al., 2012). Given that we only used four luminance (lightness-value of the ERP component), a correlation analysis was not possible. Even if differences in ERPs between mirror and non-mirror stimuli partly originated from other differences between stimuli, such as lightness or color, these differences do not convincingly explain the activation in response to a “motor action” corresponding to activity of the functional cortex.

Finally, the activated BA39, BA41, and BA42 regions are spatially close to each other and may be involved in the function of the left inferior parietal lobe in social cognition, language, and other comprehension tasks (Hauk et al., 2005; Jefferies and Lambon, 2006; Binder and Desai, 2011; Ishibashi et al., 2011; Bzdok et al., 2016). This finding supports the idea that the mirror mechanism is one of the mechanisms underlying visual–auditory interactions.

### Behavioral Results and Correlation Analysis

The significant correlation found between ERP features and speech recognition ability indicates that visual speech-related mirror stimuli better reflect the ability of speech recognition than non-mirror stimuli and visual speech-irrelevant mirror stimuli. Considering that the function of the mirror mechanism is to comprehend motor actions, this indicates that the function of visual–auditory interactions to reflect speech behavior is one function of the mirror mechanism.

Above all, the behavioral results and correlation analysis indicate that the function of visual–auditory interactions to reflect speech behavior is one function of the mirror mechanism, but that the primary cortex also participates in this function of visual–auditory interaction.

### Cross-Modal Plasticity and the Mirror Mechanism

Cross-modal plasticity is a double-edged sword in the auditory cortex regeneration of post-CI deaf people who regain auditory input. On the one hand, in the early period, cross-modal plasticity allows the visual cortex to take over the auditory cortex for functional compensation, which weakens auditory function (Finney et al., 2001; Hauk et al., 2005). On the other hand, cross-modal plasticity allows the occupied auditory cortex to regain its auditory function under auditory input *via* a CI (Anderson et al., 2017; Liang et al., 2017). Thus, it is controversial to include speech-related visual stimuli into rehabilitation training of post-CI children.

In the context of the mirror mechanism, we can also give an explanation to the dual effect to auditory function of cross-modal plasticity. In normal-hearing people, there is a balance between the executing and observing processes of a visual–auditory interaction, since they can verify whether executing (such as reading/speaking) and observing processes (such as seeing/hearing) share the same outcome of action goal (Rizzolatti et al., 2001), and auditory information benefits this process.

However, in people with hearing loss, especially those with prelingual deafness, the observing process (hearing) is absent, which causes an imbalance between executing and observing processes. This means that the patient uses the visual input (such as reading lips) as the observing process to compensate for the imbalance, which changes the outcome of the action goal into certain movements of a muscle or joint (such as lip movement). Some mirror neurons in the auditory cortex change the input of the observing process from auditory input into visual input to compensate for the absence of the observing process, which leads to a “takeover” or “inhibition” phenomenon, according to the theory of cross-modal plasticity (Finney et al., 2001; Rizzolatti et al., 2001).

Considering that the mirror mechanism is also sensitive to mastery, whereby skilled individuals have greater mirror neuron activation (Glaser et al., 2005; Calvo-Merino et al., 2006; Cross et al., 2006), the departure of auditory information and body movement (such as lip language) will be strengthened in the hearing loss period of prelingually deaf patients through repeated “training” of “comprehending” the meaning of some speech-related movements, such as lip movement (Geers et al., 2003; Burden and Campbell, 2011), for survival. Even when they regain auditory input after cochlear implantation (Giraud et al., 2001), the misleading departure is still strong. Considering the mirror mechanism sensitivity to mastery, proper rehabilitation training may change input mirror neurons to the auditory cortex from those sensitive to the observing process into an auditory input. Learning allows the auditory cortex to regain its auditory function, which leads to the phenomenon of auditory cortex recovery (weakened activation of cross-modal regions is related to improved speech performance) (Sandmann et al., 2012; Liang et al., 2017), according to cross-modal plasticity theory.

### Implications for Rehabilitation Training

For post-CI children, in the observing process, body movements to produce speech that are related to sound, such as lip reading, should be limited, since they will strengthen the misleading departure of action goal as mentioned above (Cooper and Craddock, 2006; Oba et al., 2013). However, abstract visual input to produce speech-related sound, such as words and sentences, should be promoted, since associating words or text with speech for speech comprehension is an ability of normal-hearing people and mirror neurons contribute to the comprehension of motor actions.

In the executing process, children should be trained to verify whether what they say is consistent with what they hear, which calls for the cochlear to promote the quality of auditory input and the correct of speech from others.

## Conclusion

Compared with non-mirror stimuli, mirror stimuli activated the primary auditory cortex, including BA41 and BA42, which prompted a visual–auditory interaction. Speech-related mirror stimuli (reading) activated BA39, the visual speech area, which implies the activation of mirror neurons. ERPs of the speech-related mirror stimuli group could best reflect the speech recognition ability of participants. Cross-modal plasticity is considered to underlie the correlation between visual–auditory interactions and speech recognition performance, and we hypothesized that the mirror mechanism is related to cross-modal plasticity and underlies visual–auditory interactions.

## Data Availability Statement

The raw data supporting the conclusions of this article will be made available by the authors, without undue reservation.

## Ethics Statement

The studies involving human participants were reviewed and approved by Ethics Committee of Sun Yat-sen Memorial Hospital of Sun Yat-sen University. Written informed consent to participate in this study was provided by the participants’ legal guardian/next of kin.

## Author Contributions

All authors listed have made a substantial, direct and intellectual contribution to the work, and approved it for publication.

## Conflict of Interest

The authors declare that the research was conducted in the absence of any commercial or financial relationships that could be construed as a potential conflict of interest.

## Publisher’s Note

All claims expressed in this article are solely those of the authors and do not necessarily represent those of their affiliated organizations, or those of the publisher, the editors and the reviewers. Any product that may be evaluated in this article, or claim that may be made by its manufacturer, is not guaranteed or endorsed by the publisher.
