# Supplementary material for: Mirror Mechanism Behind Visual–Auditory Interaction: Evidence From Event-Related Potentials in Children With Cochlear Implants
Source: Front Neurosci. 2021 Aug 24;15:692520. doi: 10.3389/fnins.2021.692520 (PMC8421565; doi:10.3389/fnins.2021.692520)
Supplement: Supplementary file 1 [file Data_Sheet_1.pdf]

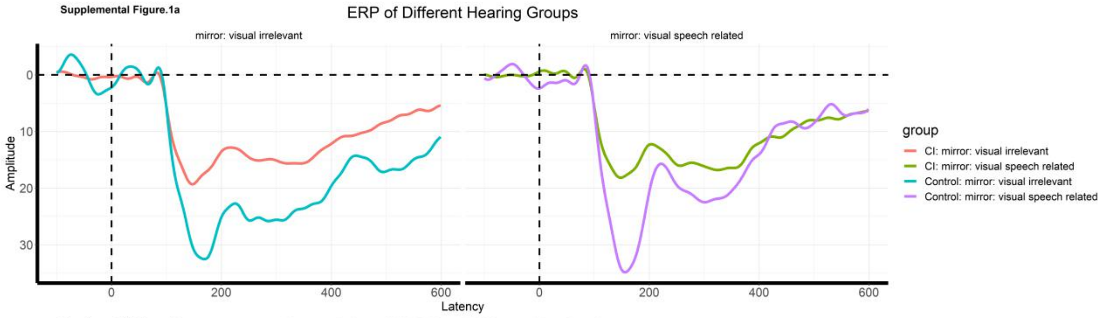

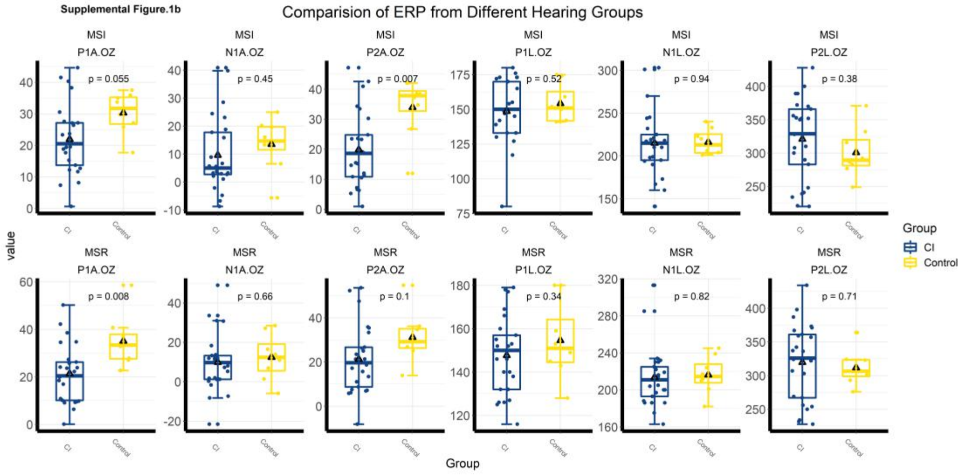

Supplemental table 1: Anova Test Result

Component	Compare_between	Df	F.value	Pr..F.
P1A.OZ	STM	1	0.07	0.8
P1A.OZ	Group	1	11.9	0*
P1A.OZ	STM:Group	1	0.7	0.41
N1A.OZ	STM	1	0	0.98
N1A.OZ	Group	1	0.7	0.41
N1A.OZ	STM:Group	1	0.03	0.86
P2A.OZ	STM	1	0.02	0.9
P2A.OZ	Group	1	9.81	0*
P2A.OZ	STM:Group	1	0.29	0.59
P1L.OZ	STM	1	0.02	0.89
P1L.OZ	Group	1	1.26	0.27
P1L.OZ	STM:Group	1	0.01	0.91
N1L.OZ	STM	1	0.02	0.88
N1L.OZ	Group	1	0.04	0.84
N1L.OZ	STM:Group	1	0.01	0.93
P2L.OZ	STM	1	0.01	0.9
P2L.OZ	Group	1	0.82	0.37
P2L.OZ	STM:Group	1	0.15	0.7

*: p.value < 0.05. Since there existing no interaction between STM & Group in each ERP component, we could do anova test on STM level with Group as factor, which was shown in Supplemental Figure.1b.

Supplemental Figure 2

CI vs Control: Mirror speech related stimuli

CI vs Control: Mirror speech irrelevant stimuli

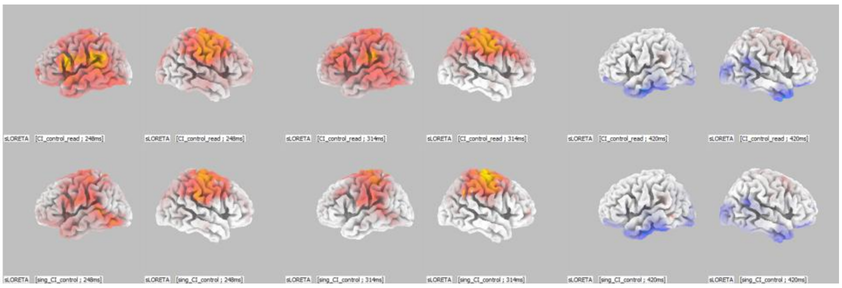

Supplemental Table 1

Groups in comparison	Component	Top5 BA of ERP difference source				
MSR	P1	40	2	13	42	22
	N1	40	3	2	5	7
	P2	36	20	35	28	34
MSI	P1	4	3	40	2	11
	N1	6	4	3	1	2
	P2	36	20	35	28	34
