# Supplementary figures and images for: Mirror Mechanism Behind Visual–Auditory Interaction: Evidence From Event-Related Potentials in Children With Cochlear Implants
Source: Front Neurosci. 2021 Aug 24;15:692520. doi: 10.3389/fnins.2021.692520 (PMC8421565; doi:10.3389/fnins.2021.692520)

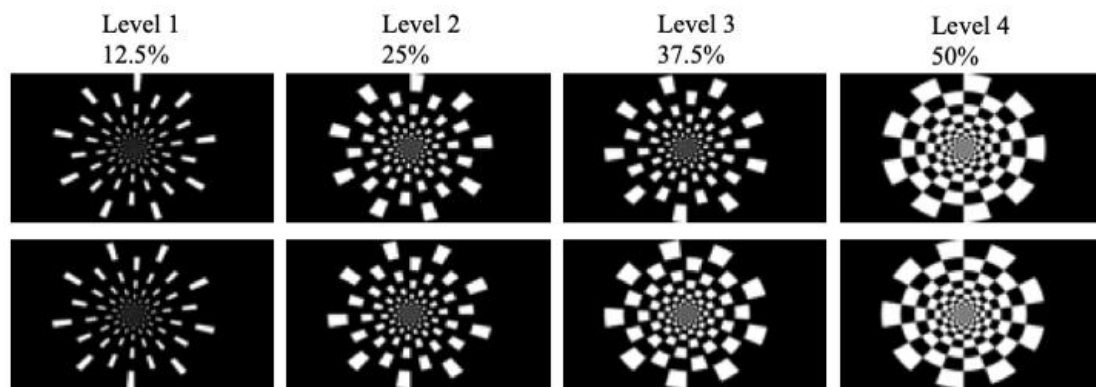

Supplementary Figure1. Checkerboard stimuli

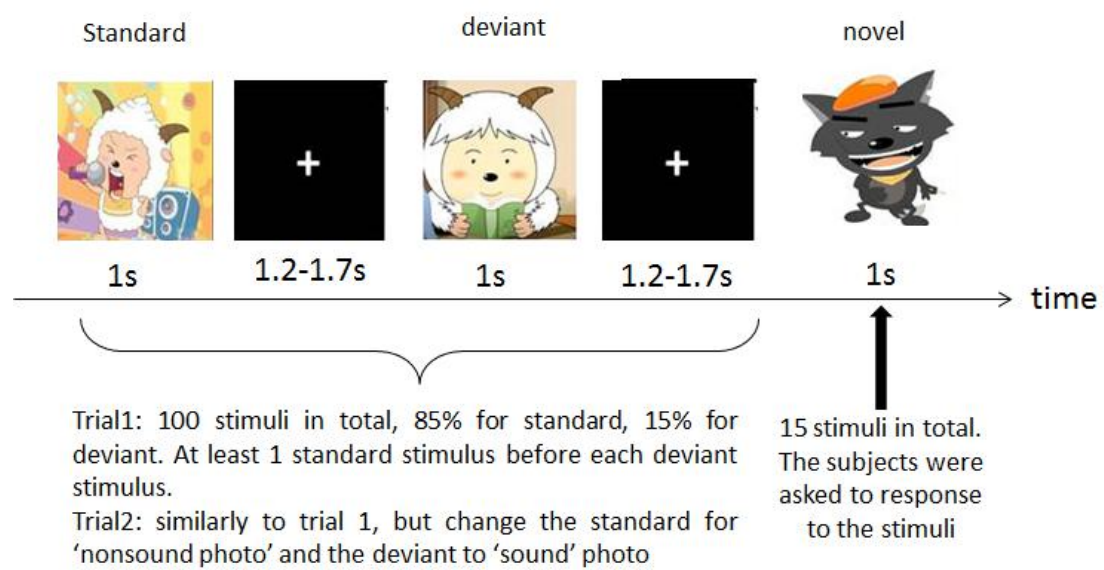

Supplementary Figure 2

Supplement: Supplementary file 2 [file Image_1.pdf]
